# LC-HRMS method for study of pharmaceutical uptake in plants: effect of pH under aeroponic condition

**DOI:** 10.1007/s11356-023-29035-1

**Published:** 2023-08-11

**Authors:** Helena Švecová, Andrea Vojs Staňová, Aleš Klement, Radka Kodešová, Roman Grabic

**Affiliations:** 1 Faculty of Fisheries and Protection of Waters, South Bohemian Research Center of Aquaculture and Biodiversity of Hydrocenoses, University of South Bohemia in České Budějovice, Zátiší 728/II, CZ-389 25, Vodňany, Czech Republic; 2 Faculty of Natural Sciences, Department of Analytical Chemistry, Comenius University in Bratislava, Ilkovičova 6, SK-842 15 Bratislava, Slovak Republic; 3 Faculty of Agrobiology, Food and Natural Resources, Department of Soil Science and Soil Protection, Czech University of Life Sciences Prague, Kamýcká 129, CZ-165 00, Prague, Suchdol, Czech Republic

**Keywords:** Pharmaceutical, Extraction, Plant uptake, Soil pollution

## Abstract

Global climate changes cause water scarcity in many regions, and the sustainable use of recycled water appears crucial, especially in agriculture. However, potentially hazardous compounds such as pharmaceuticals can enter the food chain and pose severe risks. This paper aims to study the presence of selected pharmaceutical active compounds (PhACs) and their metabolites in crops grown in aeroponic conditions and evaluate the potential of PhAC plant uptake. A solvent extraction with an acidified mixture of acetonitrile and water followed by LC-HRMS was developed and validated for quantifying nine pharmaceuticals and their nine metabolites in three plants. We aimed for a robust method with a wide linear range because an extensive concentration range in different matrices was expected. The developed method proved rapid and reliable determination of selected pharmaceuticals in plants in the wide concentration range of 10 to 20,000 ng g^−1^ and limit of detection range 0.4 to 9.0 ng g^-1^. The developed method was used to study the uptake and translocation of pharmaceuticals and their metabolites in plant tissues from an aeroponic experiment at three different pH levels. Carbamazepine accumulated more in the leaves of spinach than in arugula. On the other hand, sulfamethoxazole and clindamycin evinced higher accumulation in roots than in leaves, comparable in both plants. The expected effect of pH on plants’ uptake was not significant.

**Supplementary Information:**

The online version contains supplementary material available at 10.1007/s11356-023-29035-1.

## Introduction

Rapid urbanization and population growth directly lead to a growing demand for quality water and food, but at the same time leads to an increase in waste production, mainly wastewater (Boretti and Rosa, 2019; Sheikh Mohammad Fakhrul and Zahurul, 2019; Kookana et al., 2020). In addition, the population is aging, which is related to the increased chronic diseases and the higher consumption of different medicaments (OECD, 2022). Pharmaceutically active compounds (PhACs) in wastewater have been reported worldwide (Couto et al., 2019; Majumder et al., 2019). Not only human overuse but also drug production, food production, and improper disposal of PhACs contribute to this situation (Girotto et al., 2015; der Beek et al., 2016).

Due to global climate changes, sustainable use of recycled water affected by domestic and industrial activities, especially agriculture, appears crucial in water-scarce countries (Wu et al., 2015). Plants are often planted hydroponically under greenhouse conditions. In this case, there is also an effort to use reclaimed wastewater (Magwaza et al., 2020). The wastewater treatment process should lead to the re-entry of partially clean water into the environment but still rich in nutrients (mineral salts, phosphorous, and nitrogen) (Singh et al., 2022). However, other potentially hazardous compounds such as PhACs are usually found at ng L-1 to μg L-1 concentration levels in effluent wastewaters, but also in other environmental compartments, due to the relatively low removal efficiency of conventional wastewater treatment (Roberts and Thomas, 2006; Kümmerer, 2010; Lindberg et al., 2014; Verlicchi and Zambello, 2015). Although efficient, advanced oxidation processes used for PhAC degradation still lack economic applicability restricting their use for a high volume of effluents (Ghauch et al., 2011; Ghauch et al., 2013).

Conventionally treated wastewater is often used for crop irrigation, and residues of PhACs are found in plants (Calderón-Preciado et al., 2011; Wu et al., 2015; Al-Farsi et al., 2017; Thebo et al., 2017; Madikizela et al., 2018). So, they can enter the food chain and pose severe risks to the health of consumers and the environment (González García et al., 2018).

PhACs represent a diverse group of organic chemical substances, including prescription and over-the-counter pharmaceuticals for human and veterinary purposes. Their different chemical structures and physical-chemical properties (e.g., molecular weight, solubility, hydrophobicity, *pK*_a_, log *K*_OW_) may affect the uptake and translocation of PhACs in plants (Zhang et al., 2017). In the last few years, several studies dedicated to studying the uptake, translocation, and metabolism of xenobiotics in relatively simple and well-defined aeroponic conditions have been published (Malchi et al., 2014; Wu et al., 2014; Hurtado et al., 2016; Miller et al., 2016). Some pharmaceuticals (e.g., carbamazepine, diclofenac, sulfamethoxazole) are extensively metabolized in plant crops. Therefore, a relatively high concentration of metabolites has been found in different plant tissues/organs (Dordio et al., 2011; Pal et al., 2013; Evgenidou et al., 2015; Mackuľak et al., 2015; Cosenza et al., 2018; D’Alessio et al., 2018).

The most frequently analytical technique for studying the uptake and translocation of PhACs in plants (Eggen et al., 2011; Wu et al., 2015) is high-performance liquid chromatography hyphenated with mass spectrometry (LC-MS) of sample extracts. In the target LC-MS analysis, the most commonly used instrumentation is still a triple-quadrupole (QqQ) analyzer operated in a selected reaction monitoring mode (SRM) (Emhofer et al., 2017). Mass spectrometric detection by QqQ in SRM is sensitive but not selective enough in a heavy matrix. High-resolution mass spectrometry (HRMS) is an instrumental solution for eliminating matrix interferences and, consequently, false-positive results (Alvarez-Rivera et al., 2019). The HRMS also allows the study of the metabolism and transformation of PhACs in plant crops and the translocation of these compounds in different plant tissue (Cui and Schröder, 2016; Emhofer et al., 2017; Bigott et al., 2021).

The preparation of plant samples for target analysis of PhACs and their metabolites is based on several simple subsequent steps, including homogenization, freeze-drying, grinding, and extraction. The extraction procedure is essential for the pre-concentration of analytes, sample complexity reduction, and elimination of the matrix effect. For extraction of PhACs and their metabolites from plants, liquid extraction (LE) with simple polar solvent or solvent mixture, solid-phase extraction (SPE), accelerated solvent extraction (ASE), liquid-liquid extraction (LLE), microwave-assisted extraction (MAE), and QuEChERS (Quick, Easy, Cheap, Effective, Rugged, and Safe) were used (Kunene and Mahlambi, 2023; Herklotz et al., 2010; Matamoros et al., 2012; Zhang et al., 2013; Bartha et al., 2014; Cui and Schröder, 2016; Martínez-Piernas et al., 2018). Concerning the white chemistry concept (Nowak et al., 2021), modern sorbent or solvent-based microextraction techniques can be used (Yang et al., 2013). Published extraction procedures for plants usually used only simple solvent extraction based on acetonitrile and methanol, eventually based on the physical-chemical properties of the PhACs, and the addition of different acids, buffers, or salts (Riemenschneider et al., 2016; Montemurro et al., 2017; Martínez-Piernas et al., 2018; Pico et al., 2018). In our case, we preferred the acid condition because target analytes could be easily protonated.

This paper aimed to study the presence of selected PhACs and their metabolites in crops grown in aeroponic conditions and evaluate the potential of PhAC plant uptake. The selection of target PhACs based on our previous experience includes knowledge of ecotoxicologically relevant PhACs originated from WWTP (Golovko et al., 2014a, b; Verlicchi and Zambello, 2015; Ivanová et al., 2018). It is expected that pH of the environment, which affects ionization of compounds, can enhance or, on the contrary, limit the uptake of compounds by plants (Shahriar et al., 2022). The pH impact was described using mathematical models (Brunetti et al., 2022), but an experimental confirmation of the pH effect is rare. Therefore, we investigated the effect of pH on the uptake of 3 pharmaceuticals, which occur in the environment in different forms (e.g., cations, anions, and neutral) into plants and their subsequent translocation and transformation in two plants (spinach and arugula). We studied this effect under aeroponic conditions so that other effects, such as sorption on the soil particles, did not influence the uptake. For this purpose, we develop and validate a simple, robust, and straightforward procedure for the LC-HESI-HRMS determination of nine pharmaceuticals (with the potential to be taken up by the plants) and their primary metabolites in different plant organs/tissues.

## Materials and methods

### Chemicals

Methanol (MeOH; LiChrosolv Hypergrade) and acetonitrile (ACN; LiChrosolv Hypergrade) were purchased from Merck (Germany). Formic acid (FA) of LC/MS grade used to acidify the mobile phases and/or extraction solvent was purchased from Sigma-Aldrich (Germany). Ultrapure water (hereinafter water) was obtained from an Aqua-MAX-Ultra system (Younglin, Korea).

A list of native standards (NS) (18) and isotopically labeled (9) standards (internal standards; IS) and their producers is given in SM1. Stock solutions of all standards were prepared in methanol at a concentration of 1 mg mL^−1^ and stored in a freezer (−20 °C) for no longer than 6 months.

### Plant samples

Plants for the study were obtained from the pot experiment, where species were grown under controlled conditions (Kodešová et al., 2019a)*.* QA/QC samples (plant extracts from individual tissues) from the pot experiment were used for validation. Pea (*Pisum sativum*) was divided into leaf, stem, root, and pod. Spinach (*Spinachia oleracea*) and arugula (*Eruca sativa*) were only separated into leaf and root. Each organ from the individual plant was freeze-dried, ground to powder, and stored frozen at −20 °C until analysis.

The method was performed on the plant samples from an aeroponic experiment: spinach (*Spinacea oleracea* L., Clarinet F1) and arugula (*Eruca sativa* L., Speedy). The selection of plants was based on previously published studies by Kodesova et al. (2019a) and Kodešová et al. (2019b). The plants were grown aeroponically using a nutrient solution containing a mixture of three pharmaceuticals: carbamazepine, sulfamethoxazole, and clindamycin, with the concentration of each compound 0.1 mg L^−1^. Three scenarios with different solution pH (5, 6.5, 8) were assumed to study a pH effect on an uptake of all compounds by both plants. After 21 days, half of the plants were removed from each system, the nutrient solution was refilled, and the experiment continued for another 17 days (in total, 38 days of exposure to aeroponic conditions). Plants removed from the aeroponic systems were separated into individual tissues, freeze-dried, and homogenized. Details of the aeroponic experiment are given in SM2.

Plant tissues (spinach leaf, spinach root, arugula leaf, and arugula root) were extracted as described below. A nine-point calibration curve was prepared into the mixture of ACN:water (1:1 v/v) acidified with 0.1% FA, ranging from 0.1 to 1000 ng mL^−1^. QA/QC samples of every tissue were used for matrix standard and spiked samples. The matrix standards were prepared as a last point of calibration curve using plant extract instead of solvent. The spiked samples were extracted in the same way as unknown samples, however before extraction were spiked not only with IS (5 ng per sample) but also with NS at level 50 ng g^−1^ corresponding to 2.5-ng mL^−1^ concentration level in the extract.

### Extraction procedure

The development of the plant extraction method was based on Kodešová et al. (2019b) with some modifications. Dry plant tissue (50 mg) was weighed into the 2.0-mL Eppendorf tubes. Then five ng of IS (50 μL of IS solution in MeOH) was added and let the solvent soak and evaporate for 20 min. A stainless-steel ball and 1 mL of extraction mixture were inserted afterward. Prepared samples were homogenized for 5 min at 1800 min^−1^ (TissueLyser II, Qiagen, Germany) and centrifuged next 5 min at 10,000 min^−1^ (Mini spin centrifuge, Eppendorf). The supernatant was filtered through the 0.45-μm syringe filter from regenerated cellulose (Sartorius, Germany).

Four different extraction mixtures, (A) ACN:water (1/1 v/v) acidified with 0.1% FA, (B) ACN:water (1/1 v/v), (C) MeOH:water (1/1 v/v) acidified with 0.1% FA, and (D) MeOH:water (1/1 v/v), were used to optimize the extraction procedure for all selected pharmaceuticals and their metabolites (SM1). For this purpose, fortified samples of pea leaves were extracted in triplicates. All plant samples were spiked with NS at the concentration level of 5 ng per sample (100 ng g^−1^ dry weight). The mixture of NS was added to the dry sample just after IS and before extraction solvent addition. The best-performing extraction mixture was selected for further validation after the recovery evaluation.

### LC-HESI-HRMS analysis

LC-HESI-HRMS analysis was performed using a Q-Exactive™ HF Hybrid Quadrupole-Orbitrap™ Mass Spectrometer (Thermo Fisher Scientific, USA), coupled with a Vanquish Pumps (Dionex, Germany) and a PAL RSI autosampler (CTC Analytics AG, Switzerland). For chromatographic separation, an analytical column Hypersil Gold aQ (50 × 2.1 mm, 5 μm; Thermo Fisher Scientific, USA) was chosen with a gradient elution of mobile phase water and ACN (both acidified with 0.1% FA). A heated electrospray ionization (HESI) was used in positive ionization mode, and Q-Exactive HF operated in a high-resolution product scan (1 m/z isolation window and 15,000 FWHM resolution for product scan). LC-HESI-HRMS conditions are summarized in SM3, and MS transitions for individual compounds are given in SM1. Data acquisition was performed with Xcalibur Software, and data were processed by TraceFinder 3.3 Software (Thermo Fisher Scientific).

The performance characteristics of the method were evaluated for the tissues from three plants (pea, spinach, arugula) and 18 relevant pharmaceuticals and their metabolites. Internal standard and matrix-matching standard methods were used to quantify target analytes (Grabicova et al., 2018). Response factor (*RF*), average response factor (*ARF*), and concentration of target analytes were calculated as described elsewhere (Borik et al., 2020a; Borik et al., 2020b). Recoveries were assessed for each matrix as a heptaplicate analysis of fortified control experiment samples (irrigated with drinking water only). The extraction efficiency of the procedure was evaluated at two low concentration levels (10 and 100 ng g^−1^) and relatively high concentrations corresponding to a concentration of PhACs and their metabolites in the aeroponic experiment (1000 ng g^−1^). Matrix effect (ME) was calculated as follows: (*RF*_MST_ − *ARF*)/*ARF* × 100%, where the difference between the average *RF* of calibration standards and *RF* of matrix-matched standards (*RF*_MST_) exceeded 20% and *RF*_MST_ was used to quantify the target analyte in target tissue instead of *ARF*. The limit of quantification (LOQ) was calculated for each compound in each sample. Only calibration points with a relative standard deviation (RSD) of less than 30% deviation from *ARF* were used (Grabicova et al., 2018). The peak area of the lowest calibration point divided by a factor of 2 was substituted for quantification instead of the peak area in corresponding calculation. This approach resulted in unique LOQ dataset corrected to IS recovery and matrix effect across the samples and matrices.

## Results and discussion

### Extraction method selection

We tested four extraction solvent mixtures: (A) ACN:water (1/1 v/v) acidified with 0.1% FA, (B) ACN:water (1/1 v/v), (C) MeOH:water (1/1 v/v) acidified with 0.1% FA, and (D) MeOH:water (1/1 v/v). As can be seen from Table 1, all solvents have shown similar recovery, and no evident differences were observed across tested mixtures. All recovery values range from 60 to 130%, except 10,11-epoxide CBZ in solvent C. Mean recoveries (and RSD in brackets) obtained for target analytes were 102% (8%), 99% (4%), 102% (4%), and 97% (3%) for solvents A, B, C, and D, respectively. Such differences between all four solvent mixtures are negligible, contrary to our experience with fish tissues (Grabicova et al., 2018). Therefore, solvent A was selected for further evaluation due to the similar composition of mobile phases used in subsequent LC-HESI-HRMS analysis.

**Table 1:** Recoveries for all pharmaceuticals in pea leaf for different extraction solvents

Pharmaceutical	Recovery ± RSD (%)			
	Solvent A	Solvent B	Solvent C	Solvent D
Atenolol	110 ± 9	112 ± 4	105 ± 1	99 ± 2
Carbamazepine (CBZ)	101 ± 8	94 ± 1	104 ± 0	98 ± 1
10,11-Epoxide CBZ	104 ± 12	76 ± 6	131 ± 5	103 ± 2
10,11-Dihydro CBZ	110 ± 8	98 ± 3	110 ± 2	106 ± 4
10,11-Dihydro dihydroxy CBZ	85 ± 10	127 ± 13	102 ± 14	88 ± 9
Citalopram	102 ± 7	97 ± 2	99 ± 3	94 ± 2
Clarithromycin	109 ± 10	108 ± 1	96 ± 1	96 ± 2
Clindamycin	107 ± 8	86 ± 4	121 ± 6	116 ± 3
Clindamycin sulfoxide	84 ± 9	82 ± 5	84 ± 3	86 ± 3
Fexofenadine	90 ± 5	85 ± 3	103 ± 1	97 ± 0
Irbesartan	91 ± 7	112 ± 4	97 ± 2	98 ± 2
Metoprolol	110 ± 8	94 ± 2	103 ± 2	99 ± 2
Metoprolol acid	99 ± 8	79 ± 5	96 ± 2	91 ± 1
N1-acetylsulfamethoxazole	137 ± 11	115 ± 5	90 ± 11	80 ± 1
N4-acetylsulfamethoxazole	102 ± 4	112 ± 4	96 ± 4	95 ± 5
N-desmethylcitalopram	88 ± 6	100 ± 1	96 ± 3	89 ± 1
Oxcarbazepine	91 ± 10	102 ± 2	93 ± 2	88 ± 2
Sulfamethoxazole	110 ± 8	97 ± 1	110 ± 2	116 ± 3

Samples were spiked at concentration level 100 ng g^−1^

### Validation of method

Method validation was performed to evaluate the linearity, LOQ, precision, and trueness (Kruve et al., 2015).

Linearity was tested using nine-point calibration curve (0.1, 0.5, 1, 5, 10, 50, 100, 500, and 1000 ng mL^−1^) and was expressed as the ratio NS to IS peak area depending on concentration (Borik et al., 2020a). Most substances showed excellent linearity from 0.5 to 1000 ng mL^−1^ (squares of residues *r*^2^ > 0.99). ATE, 10,11-epoxide CBZ, and oxcarbazepine showed higher values (*r*^2^ > 0.99) but only from 0.5 to 500 ng mL^−1^. Linear response over 4 orders of magnitude is necessary because an extensive range of concentration in different matrices was expected in the experiments. Considering the sample amount (50 mg) and extract volume (1 mL), the method was linear in the range of 10 to 20,000 ng g^−1^. LOQs of the method were determined according to Grabicova et al. (2018) and Borik et al. (2020a) and applying criteria described in the “LC-HESI-HRMS analysis” section. Table 2 presents a range of LOQ value overall validation set of samples from eight matrices. Calculated LOQ values are lower than those reported in similar studies (Carter et al., 2014; Klement et al., 2020; Brunetti et al., 2021). LOQs varied from low units of ng g^−1^ to 27 ng g^−1^. Among studied compounds, both SUL metabolites showed the highest LOQs, which can be assigned to their relatively low response in HESI. A relatively wide LOQ range for individual compounds indicates a high matrix effect for some plant tissues.

**Table 2:** Limit of quantification (LOQ) and limit of detection (LOD) expressed as the minimal and maximal values found for the target compound in all plant organs (pea — leaf, stem, pod, root; arugula — leaf, root; spinach — leaf, root)

	LOQ		LOD	
	Min (ng g^−1^)	Max (ng g^−1^)	Min (ng g^−1^)	Max (ng g^−1^)
Atenolol	2.4	7.9	0.80	2.6
Carbamazepine (CBZ)	2.2	15	0.73	5.0
10,11-Epoxide CBZ	1.2	13	0.40	4.3
10,11-Dihydro CBZ	4.6	14	1.5	4.7
10,11-Dihydro dihydroxy CBZ	2.8	17	0.93	5.7
Citalopram	4.2	7.1	1.4	2.4
Clarithromycin	4.6	27	1.5	9.0
Clindamycin	2.3	6.9	0.77	2.3
Clindamycin sulfoxide	1.8	11	0.60	3.7
Fexofenadine	2.3	14	0.77	4.7
Irbesartan	4.1	7.5	1.4	2.5
Metoprolol	3.3	9.9	1.1	3.3
Metoprolol acid	1.9	9.6	0.63	3.2
N1-acetylsulfamethoxazole	8.1	27	2.7	9.0
N4-acetylsulfamethoxazole	7.6	21	2.5	7.0
N-desmethylcitalopram	3.4	7.3	1.1	2.4
Oxcarbazepine	5.7	14	1.9	4.7
Sulfamethoxazole	6.7	14	2.2	4.7

**Fig. 1:**
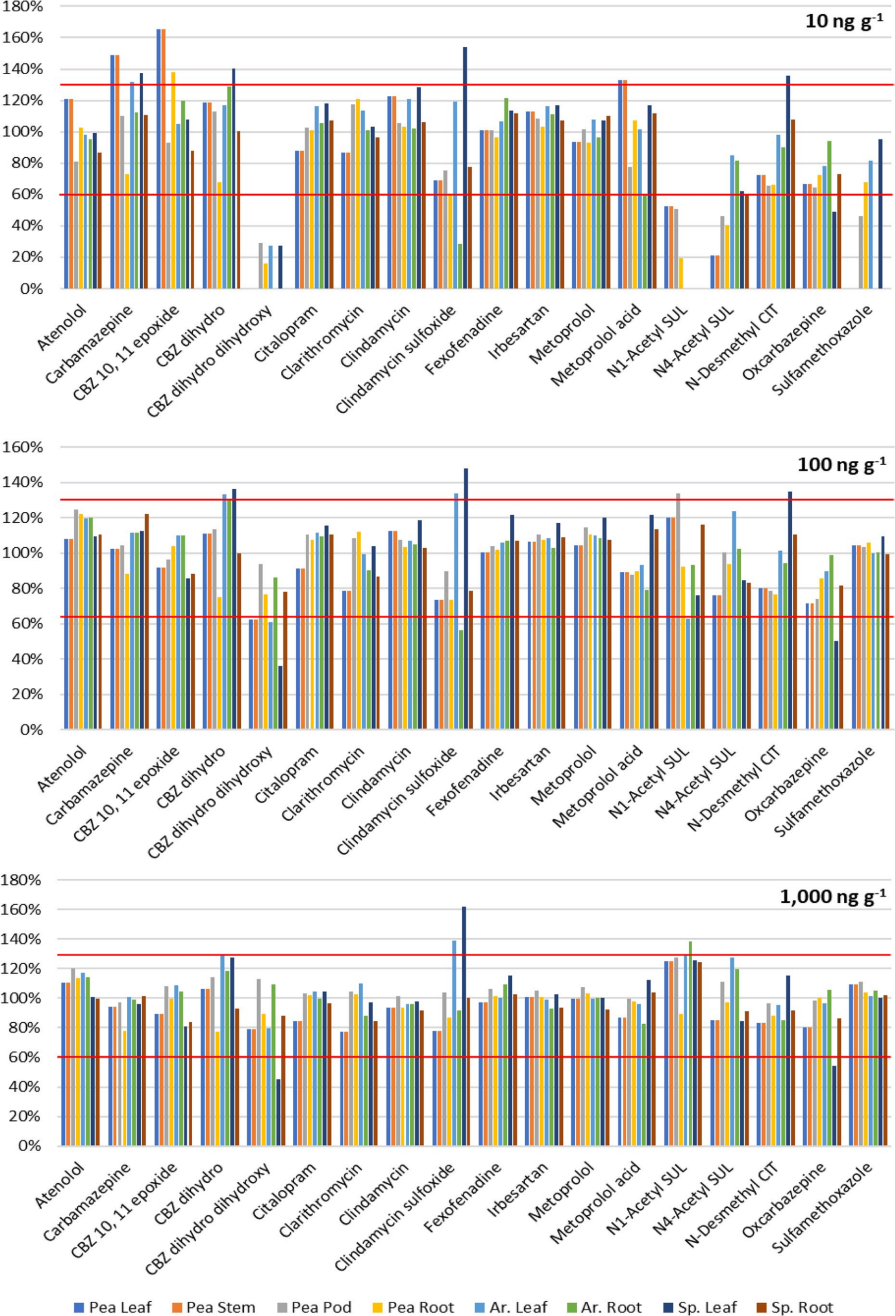
Average recoveries for eight plant tissues and 18 tested compounds at three different concentration levels (10, 100, 1000 ng per g of dry tissue). Red lines border interval from 60 to 130%

The recovery for 18 compounds at three concentrations levels — 10, 100, and 1000 ng per g of dry weight — for eight different plant tissues was evaluated. The recoveries for level 1000 ng g^−1^ ranged from 85 to 123%, and for level 100 ng g^−1^ ranged from 70 to 115%, respectively. For the lowest level (10 ng g^−1^), recoveries ranged from 71 to 123%, with an exception for 10,11-dihydro dihydroxy CBZ (25%), *N1*-acetyl SUL (44%), and *N4*-acetyl SUL (52%). Another study also observed low recovery for SUL in different plant leaves (Goldstein et al., 2014). As shown in Fig. 1, only a small number of individual recovery values are out of the acceptable range (60–130%) for all tested concentration levels in all matrices. Most of the overestimated values are related to green parts of plants (leaf of spinach above other leaves — see SM4) in contrast to root samples, where only a few cases of recoveries below 60% were observed (the root of arugula, pea, and spinach, respectively).

Heptaplicates of fortified samples were used not only for recovery evaluation but also for estimating method precision expressed by RSD (calculated for each compound, tissue, and concentration level) (Taverniers et al., 2004; Kruve et al., 2015). RSD for heptaplicates at the lowest level of 10 ng g^−1^ ranged from 0 to 24% (median value is 5%). As mentioned above, the method showed the worst performance for 10,11-dihydro dihydroxy CBZ, *N1*-acetyl SUL, and *N4*-acetyl SUL. This finding relates to the above-described relatively low HESI response resulting in high LOQs. Also, 10,11-epoxide CBZ shows higher RSD for this concentration level. The RSDs at the concentration level 100 ng g^−1^ ranged from 1 to 14% (except for *N1*-acetyl SUL in arugula and spinach leaf) with a median of 3%, and for the highest validated level (1 μg g^−1^) ranged from 1 to 8% with the median value of 2%.

**Fig. 2:**
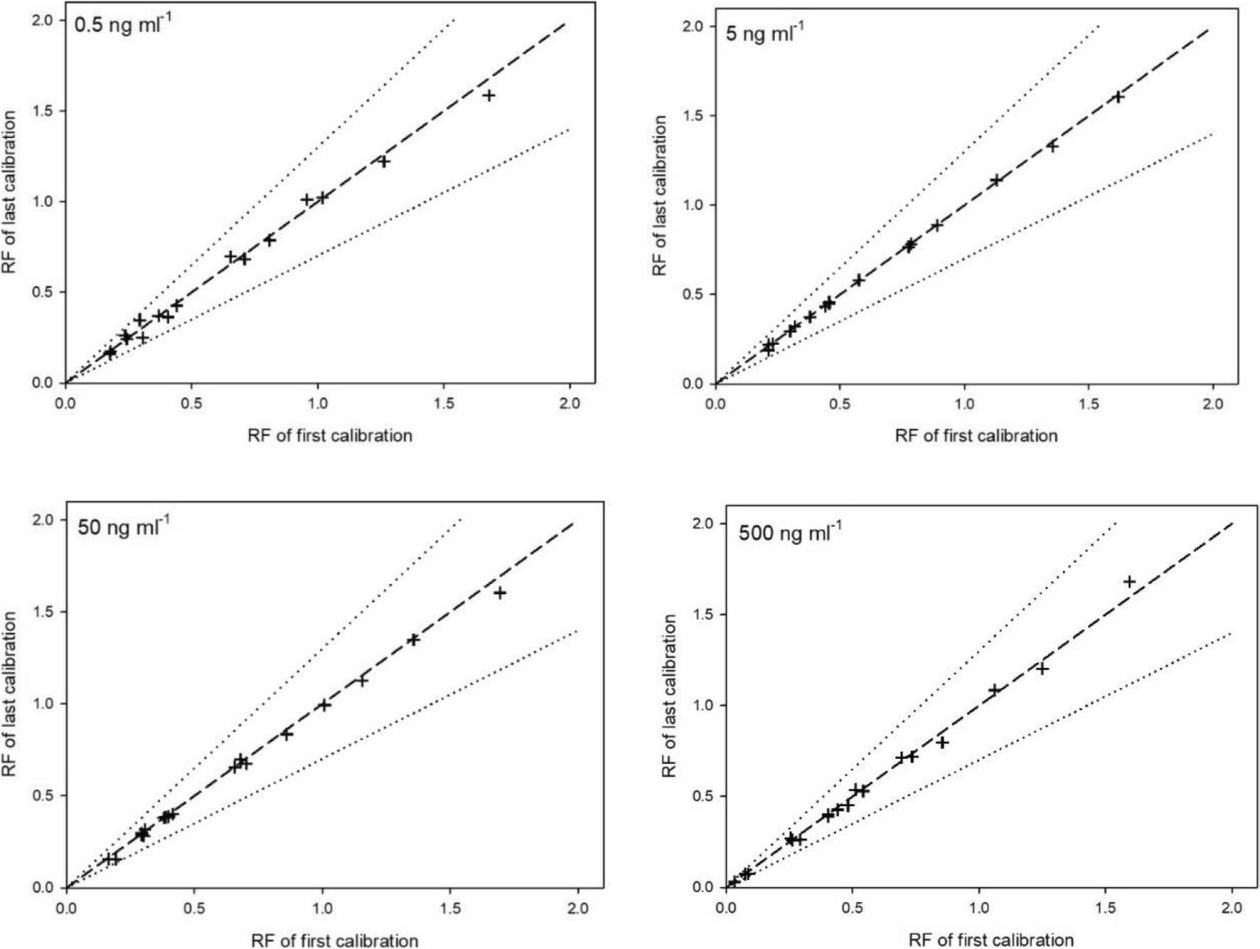
Stability of relative response factor at four different concentration levels in the sequence of 170 measured samples (pea leaf, stem, pod, and root). The dashed line shows the ideal fit, and the dotted lines represent a 30% confidence interval

The stability of the analytical signal (robustness of the detection) was investigated as a parameter that strongly influences quantification. The stability of the *RF* over time, which can relate to the stability of high-resolution product scan (HRPS) detection (Grabicova et al., 2018), was evaluated during the sequence of 170 analysis runs (around 43 h of measurement time). As shown in Fig. 2, relative response factors for the first and last calibration fit almost perfectly. This graph shows the method’s robustness for routinely analyzing many miscellaneous plant tissue samples.

### Matrix effects

MEs were evaluated by comparing *ARF* from calibration with *RF* of matrix-matched standards (*RF*_MST_) and are reported in SM5. Negative numbers mean signal suppression in contrast to enhancement represented by positive MEs.

From the tissue point of view, there was only slight variation among the green parts of a plant and its roots. Seven target compounds from 18 were calculated using a matrix matching standard for pea stem, unlike pea root (five compounds from 18). The same trend was also found for arugula leaf (6/18) and root (3/18) or spinach leaf (5/18) and root (2/16). Ion enhancement higher than 30% was observed for 10,11-dihydro dihydroxy CBZ (arugula and spinach leaf), metoprolol acid, and *N*-desmethyl CIT (both in pea tissues). Ion suppression lower than −30% was observed for 10,11-dihydro dihydroxy CBZ and *N1*-acetyl SUL in pea tissues, and clarithromycin, clindamycin sulfoxide, metoprolol acid, and oxcarbazepine in arugula and spinach tissues (Goldstein et al., 2014). Generally, the highest matrix effect was observed for compounds showing the worst performance (SM5).

### Effect of pH on pharmaceutical uptake under aeroponic condition

A developed plant extraction and analysis method was applied to plant samples from the aeroponic experiment. During this experiment, spinach and arugula were grown aeroponically using the only solution of pharmaceuticals of interest, nutrients, and pH additives. Therefore, soil properties did not influence PhAC uptake in plants, which is the main advantage of aeroponics (Madikizela et al., 2018).

**Fig. 3:**
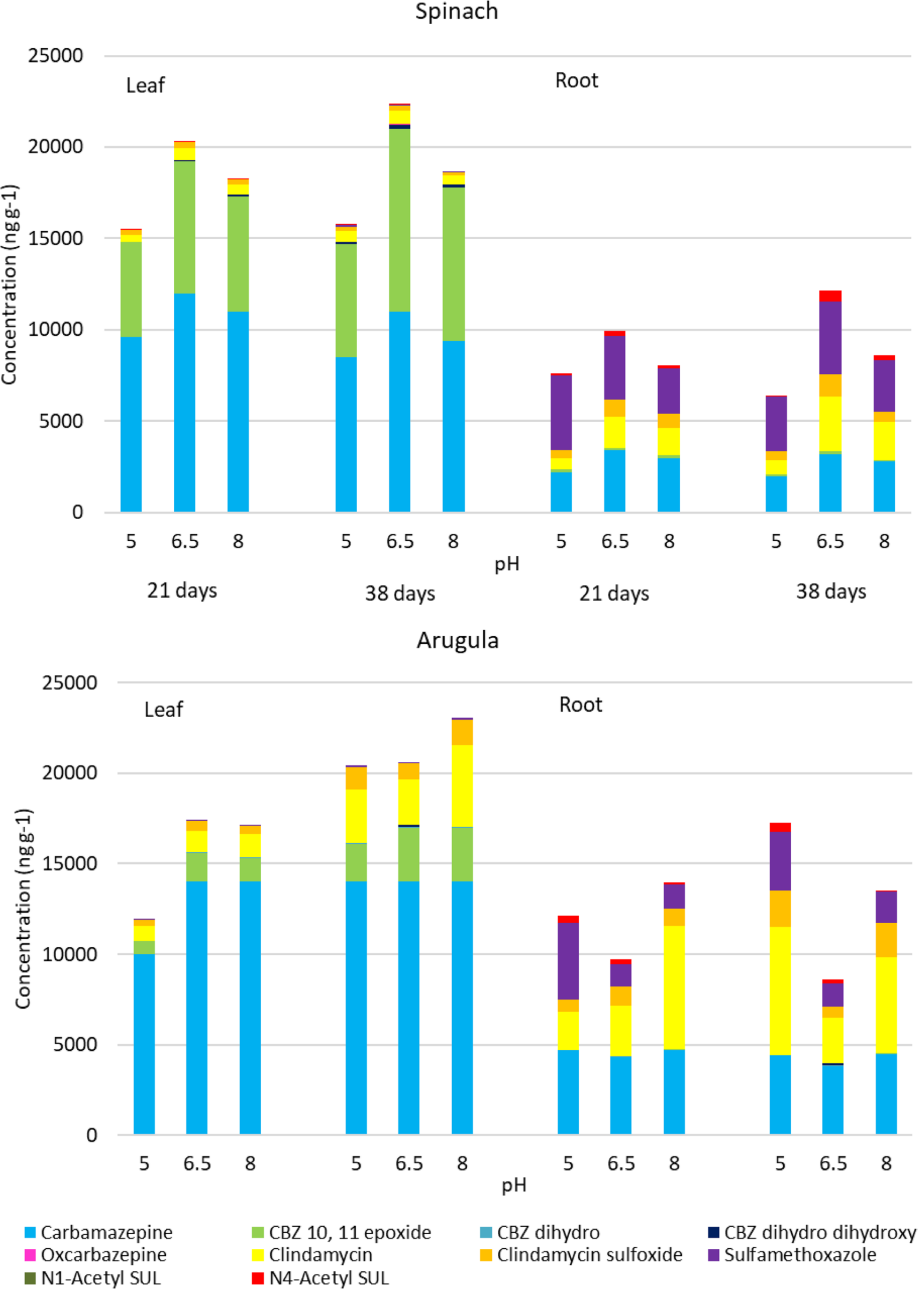
Pharmaceuticals and their metabolite concentration in spinach and arugula during aeroponic experiments under different pH conditions (5, 6.5, 8)

Results of plant tissues analysis are reported in supplementary materials SM6 (including recoveries in SM7) and visualized in Fig. 3. Concentrations of CBZ and its metabolites in the roots and leaves of both plants indicate that CBZ is a highly mobile compound in plant bodies due to the neutral form of its molecule, low molecular weight, low lipophilicity, and low number of H-bonds. CBZ accumulates mainly in plant leaves, i.e., at the end of a transpiration stream (Ben Mordechay et al., 2018; Brunetti et al., 2019; Kodesova et al., 2019a; Kodešová et al., 2019b; Brunetti et al., 2021). CBZ is a relatively stable compound in the water environment, but it can be metabolized in plants (mainly in leaves), which is attributed to plant cytochrome P450 enzymes (Goldstein et al., 2014; Malchi et al., 2014; Ben Mordechay et al., 2018; Kodesova et al., 2019a; Kodešová et al., 2019b). Ratios between concentrations of CBZ and its metabolites (especially 10,11-epoxide CBZ) in leaves proved our previous findings (Kodešová et al., 2019b) that the degree of the CBZ transformation in plants depends on the plant family. Our study again proved that the efficiency of arugula (family *Brassicaceae*) in metabolizing is low (i.e., the CBZ fraction is much more significant than the fraction of 10,11-epoxide CBZ) in comparison to the moderate efficiencies of spinach (i.e., comparable concentrations of CBZ and 10,11-epoxide CBZ).

Concentrations of SUL and its metabolite in plant tissues show that this compound is mainly accumulated in roots. It can be explained by mostly negatively charged molecules, i.e., repulsion between their molecules and negatively charged cell walls (Kodešová et al., 2019b). Another reason can be the transformation of SUL in plant tissues (Brunetti et al., 2022). Our data do not indicate considerable differences between the uptake, transition, and transformation observed for both plants.

Finally, concentrations of CLI and its metabolite show a more considerable accumulation of both compounds in roots than in leaves. This finding can be explained by the primarily positive charge of the CLI molecules and reduced uptake and translocation of this compound due to its sorption onto the negatively charged cell walls (Brunetti et al., 2022). Results also show that CLI and its metabolite concentrations were higher in the arugula tissues than in the spinach tissues. It may suggest that arugula plants are more efficient in the uptake and translocation of this compound than spinach plants. However, because it can be assumed that CLI can be metabolized in plant bodies (Brunetti et al., 2022), it can also be hypothesized that spinach’s efficiency in transforming CLI and its metabolite is similar to CBZ compounds, much greater than that of arugula.

Regarding the impact of pH on the compounds’ uptake by plants, it was presumed that pH, which affects forms of ionizable compounds, should influence their uptake by plants. As discussed above, while plants should quickly take up neutral compounds, uptake of the ionized compound should be restricted due to either their sorption onto cell walls (cations) or repulsion from the cell walls (anions). Based on these presumptions, the behavior of the CBZ molecule, which was over the entire pH range in the neutral form, and its uptake should not be influenced by the pH of a solution. The *pK*_a_ value (strongest acidic) for SUL is 6.16. Thus, at a solution pH of 5, this compound was partly in neutral form, which could increase its uptake by plant roots. This effect may explain a higher concentration of SUL in the roots of arugula at a pH of 5 than at other pH conditions. However, a similar effect is not visible in the case of spinach. The *pK*_a_ value (the strongest basic) for CLI equals 7.55. Thus, at pH 8 compound was partly in neutral form, which could increase its uptake by plant roots. Such effect can be identified for arugula roots harvested on the 21st day but not for arugula roots harvested on the 38th day. In addition, this effect is not evident at all for spinach. Another factor that could affect the accumulation of compounds in plants could be the plant growth that was impacted by solution pH. For instance, in the case of spinach, the largest sum of compounds’ concentrations (especially concentrations of CBZ and its metabolite) in plant tissues was observed at pH of 6.5, followed by those at pH of 8 and 5 (Fig. 3). This trend corresponds to trends in the areas of plant roots and leaves (Fig. SM2.4) and partly also to trends in their masses (Fig. SM2.3). Those conditions for plant growth (aggravated by too low or too high pH or even by a complex of compounds in solution) reduced the growth of plants as well as their transpiration. They thus reduced compounds’ accumulation in plant tissues. Finally, it can also be assumed that the uptake of all compounds could be affected by their mutual interactions and interactions with other components in the solution-plant system. However, there is not enough information available for this assessment. In addition, our previous studies (Kodešová et al., 2019b; Klement et al., 2020) did not find an influence of a mixture of different compounds on their uptake from soils.

## Conclusion

In our study, a robust, fast, and reliable extraction procedure followed by LC-HESI-HRMS was developed, optimized, and applied to study pharmaceutical uptake to plants. The simple solvent extraction with an acidified mixture of acetonitrile and water (0.1% FA) was selected as the optimal extraction solvent for four different plant tissues. Subsequent LC-HESI-HRMS analysis was optimized and validated for 4 orders of magnitude range of pharmaceutically active compounds in eight plant matrices. Finally, the suitability of the validated method was confirmed for 18 compounds with various physical-chemical properties and potential to plant uptake under different pH conditions in a wide concentration range.

Some substances are taken up by plants and further metabolized. Our method for PhAC detection and quantification was used in an aeroponic experiment, where soil properties could not influence PhAC uptake in plants. In this simplified model of plant cultivation, carbamazepine proved higher accumulation and metabolization in leaves than in roots, unlike sulfamethoxazole and clindamycin, which accumulate more in roots. Arugula, as a representative of the family *Brassicaceae*, has confirmed a low ability to metabolize CBZ, compared to this ability of other plants like spinach. The expected positive effect of the modified charge of both ionic compounds, due to pH adjustment, on their uptake by plants was likely masked/reduced by the negative pH influence on plant growth and transpiration intensity, i.e., on the intensity of the solution uptake by roots.

In conclusion, the analytical method developed in our study can improve the possibility of gaining relevant results from experiments dealing with PhACs’ uptake in plants.

## Supplementary information

ESM 1

## Data Availability

Not applicable.
