# Supplementary material for: LC-HRMS method for study of pharmaceutical uptake in plants: effect of pH under aeroponic condition
Source: Environ Sci Pollut Res Int. 2023 Aug 11;30(42):96219–30. doi: 10.1007/s11356-023-29035-1 (PMC10482775; doi:10.1007/s11356-023-29035-1)
Supplement: Supplementary file 1 — ESM 1 [file 11356_2023_29035_MOESM1_ESM.docx]

**Pharmaceuticals uptake in plants: Effect of pH under aeroponic condition**

***Supplementary materials***

Helena Švecová^a,*^, Andrea Vojs Staňová^a,b^, Aleš Klement^c^, Radka Kodešová^c^, Roman Grabic^a^

^a^ University of South Bohemia in České Budějovice, Faculty of Fisheries and Protection of Waters, South Bohemian Research Center of Aquaculture and Biodiversity of Hydrocenoses, Zátiší 728/II, CZ‑389 25 Vodňany, Czech Republic

^b^ Comenius University in Bratislava, Faculty of Natural Sciences, Department of Analytical Chemistry, Ilkovičova 6, SK‑842 15 Bratislava, Slovak Republic

^c^ Czech University of Life Sciences Prague, Faculty of Agrobiology, Food and Natural Resources, Dept. of Soil Science and Soil Protection, Kamýcká 129, CZ-165 00 Prague - Suchdol, Czech Republic

*Corresponding author: E-mail: [hsvecova@frov.jcu.cz](mailto:hsvecova@frov.jcu.cz) (H. Švecová)

**SM1:** Compounds – producers and MS transitions used in HPLC-HESI-HRMS analysis of plant extracts.

| **Analyte** | **Producer** | **Used IS** | **Parent ion** | **Quan** | **Qual** | **NCE** | **RT (min)** | **pKa^a^** |
| --- | --- | --- | --- | --- | --- | --- | --- | --- |
| Atenolol (ATE) | Sigma Aldrich | Atenolol_IS | 267.1703 | 190.0863 | 145.0645 | 60 | 3.18 | 9.67 B, 14.08 A |
| Atenolol_IS, D6 | Alsachim | - | 273.2080 | 145.0653 | - | 60 | 3.18 |  |
| Carbamazepine (CBZ) | Sigma Aldrich | CBZ_IS | 237.1022 | 194.0964 | 192.0808 | 50 | 5.96 | -3.8 B, 15.96 A |
| CBZ_IS, D10 | Chiron | - | 247.1650 | 204.1593 | - | 50 | 5.96 |  |
| CBZ 10,11 epoxide | TRC | CBZ_IS | 253.0972 | 210.0913 | 180.0811 | 25 | 5.30 |  |
| CBZ dihydro | TRC | CBZ_IS | 239.1179 | 194.0965 | 222.0913 | 45 | 6.02 |  |
| CBZ dihydro dihydroxy | TRC | CBZ_IS | 271.1177 | 254.0813 | 210.0915 | 25 | 4.66 |  |
| Citalopram (CIT) | AK Scientific | CIT_IS | 325.1711 | 109.0451 | 262.1025 | 50 | 6.08 | 9.78 B |
| CIT_IS, D6 | TRC | - | 331.2085 | 109.0454 | - | 50 | 6.08 |  |
| *N*-desmethyl CIT | LGC | CIT_IS | 311.1554 | 109.0450 | 262.1024 | 35 | 6.00 |  |
| Clarithromycin (CLA) | Sigma Aldrich | Clarithromycin_IS | 748.5000 | 158.1176 | 590.3900 | 15 | 6.58 | 9 B, 12.46 A |
| Clarithromycin_IS, D3 | TRC | - | 751.5030 | 161.1367 | - | 15 | 6.58 |  |
| Clindamycin (CLI) | Sigma Aldrich | Clindamycin_IS | 425.1871 | 126.1278 | 377.1638 | 35 | 5.21 | 7.55 B, 12.41 A |
| Clindamycin_IS, D3 | TRC | - | 428.2060 | 129.1469 | - | 35 | 5.21 |  |
| Clindamycin sulfoxide | TRC | Clindamycin_IS | 441.1821 | 377.1835 | 126.1279 | 20 | 4.52 |  |
| Fexofenadine (FEX) | TRC | Fexofenadine_IS | 502.3000 | 466.2742 | 484.2846 | 40 | 6.66 | 9.01 B, 4.04 A |
| Fexofenadine_IS, D6 | TRC | - | 508.3324 | 177.1545 | 472.3114 | 40 | 6.66 |  |
| Irbesartan (IRB) | TRC | Irbesartan_IS | 429.2000 | 207.0917 | 195.1490 | 35 | 6.42 | 4.12 B, 5.85 A |
| Irbesartan_IS, D4 | TRC | - | 433.3644 | 211.1165 | 195.1491 | 35 | 6.42 |  |
| Metoprolol (MET) | Sigma Aldrich | Metoprolol_IS | 268.1907 | 116.1074 | 159.0805 | 50 | 4.57 | 9.67 B, 14.09 |
| Metoprolol_IS, D7 | Alsachim | - | 275.2346 | 123.1513 | - | 50 | 4.57 |  |
| Metoprolol acid | TRC | Atenol_IS | 268.1543 | 145.0648 | 191.0702 | 40 | 3.80 |  |
| Oxcarbazepine | Sigma Aldrich | CBZ_IS | 253.0972 | 236.0707 | 208.0757 | 30 | 5.48 | -4.3 B, 13.18 A |
| Sulfamethoxazole (SUL) | Sigma Aldrich | SUL_IS | 254.0594 | 156.0112 | 108.0447 | 45 | 4.74 | 1.97 B, 6.16 A |
| SUL_IS, 13C6 | HPC | - | 260.0795 | 162.0317 | - | 45 | 4.74 |  |
| *N1*-Acetyl SUL | Sigma Aldrich | SUL_IS | 296.0699 | 254.0585 | 156.0111 | 10 | 5.72 |  |
| *N4*-Acetyl SUL | Sigma Aldrich | SUL_IS | 296.0699 | 198.0221 | 134.0600 | 30 | 5.08 |  |

Quan = quantitative ion; Qual = qualitative (confirmation) ion; RT = retention time

All purchased chemicals were of high purity(> 98%);

TRC … Toronto Research Chemicals (Canada), HPC … HPC Standards GmbH (Germany), LGC (United Kingdom), Alsachim (France), AK Scientific (USA), Chiron Chemicals (Australia)

IS… isotopically labeled internal standard

^a^ … predicted pKa values – strongest acidic (A) and strongest basic (B) searched from https://go.drugbank.com, 9th February 2023

**SM2:** Aeroponics experiment

Two plants were selected: spinach (*Spinacea oleracea* L., Clarinet F1) and arugula (*Eruca sativa* (L.), Speedy). Selection was based on results of our previous studies by Kodešová et al. (2019a,b) that showed that while spinach plants can efficiently metabolize carbamazepine in their leaves, arugula plants are much less efficient. The plants were grown aeroponically using the twelve (6 for each plant) aeroponic systems AERO-STAR 40 (Platinium). Each system includes 4 pots above their own 16-liter tank with the nutrient solution (**Fig. SM2.1**). A drip and a rotating sprayer for each of the pots was used to continuously distribute the nutrient solution, saturated with oxygen throughout the root chamber. The roots were thus constantly in contact with the solution, similar to a standard hydroponic system. However, water was not stagnant and was enriched with oxygen. Seeds of plants were first germinated in a horticultural substrate and, after sprouting, were moved into the pots (3 plant per each pot). After 21 days a half of plants were removed from each system, nutrient solution was refiled and experiment continued for other 17 days (i.e., in total 38 days of exposure to aeroponic conditions).

The 16-liter nutrient solutions applied for each system contained initially and after refiling 5 mL of fertilizer BioSevia (General Hydroponics). The nutrient solutions for 3 systems for each plant also contained a mixture of 3 pharmaceuticals carbamazepine, sulfamethoxazole and clindamycin. The intended concentration of each compound was 0.1 mg L^-1^. In addition, each nutrient solution also contained potassium bicarbonate and citric acid, which were used to adjust nutrient solution pH at 5.0, 6.5 and 8.0 (i.e., different pH for 3 system without and with the mixture of pharmaceuticals, respectively, for each plant). Solution pH and salinity was periodically measured using the Combo pH & EC tester (Hanna Instruments) and adjusted if necessary.

Experiment was carried out in the greenhouse with restricted natural light and with air-condition. GIB lighting Growth Spectrum Advanced lamps with a power of 600 W were used as a light source (**Fig. SM2.2**). Growing shades Adjust-A-Wings were used to make efficient use of the light and heat generated by the emitter. The light mode maintained by this system had a frequency of 12 hours of illumination and 12 hours of darkness. A temperature during the day and at night was set at 24°C and 18°C, respectively. Air humidity varied between 30 and 40%.

Plants removed from the aeroponic systems were divided into separate tissues, scanned and weighted (i.e., wet masses). Next, plant tissues were freeze-dried, and the dry masses were evaluated. All samples were ground and pooled (i.e., each sample of roots or leaves collected 21^st^ day and 38^th^ day represented one aeroponic system). All samples were stored under -21°C until analyzed.

The wet and dry masses of plant tissues, and tissue dry mass characterized plants growth under different conditions (**Fig. SM2.3**). Another indicator of plant growth was the area of roots and leaves, which was analyzed on the images of plant tissues using the ImageJ software version 1.52a (Rasband W, 1997-2019) and the Color threshold function. Finally, the ration between wet or dry masses and areas of tissues were calculated to assess physiological conditions of plant tissues (**Fig. SM2.4**).

Kodešová, R., Klement, A., Golovko, O., Fér, M., Nikodem, A., Kočárek, M., Grabic, R., 2019a. Root uptake of atenolol, sulfamethoxazole and carbamazepine, and their transformation in three soils and four plants. Environ. Sci. Pollut. Res., 26, 10, 9876–9891. https://doi.org/10.1007/s11356-019-04333-9

Kodešová, R., Klement, A., Golovko, O., Fér, M., Kočárek, M., Nikodem, A., Grabic, R., 2019b. Soil influences on uptake and transfer of pharmaceuticals from sewage sludge amended soils to spinach. J. Environ. Manage., 250, 109407. https://doi.org/10.1016/j.jenvman.2019.109407

Rasband, W.S., 1997–2014. ImageJ, U. S. National Institutes of Health, Bethesda, Maryland, USA, http://imagej.nih.gov/ij/.


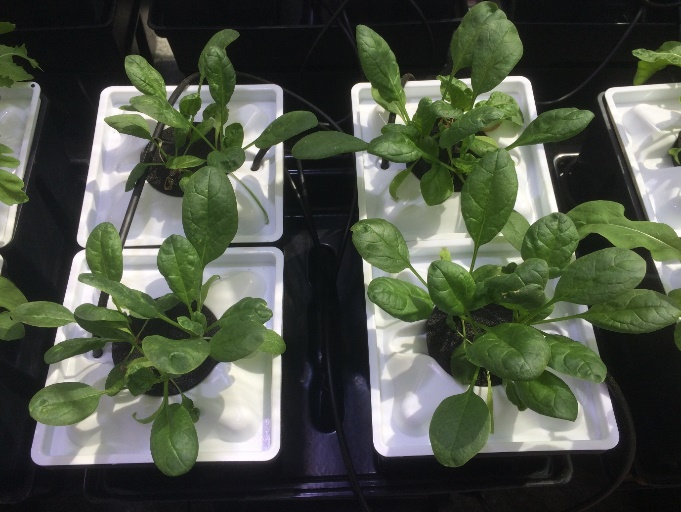

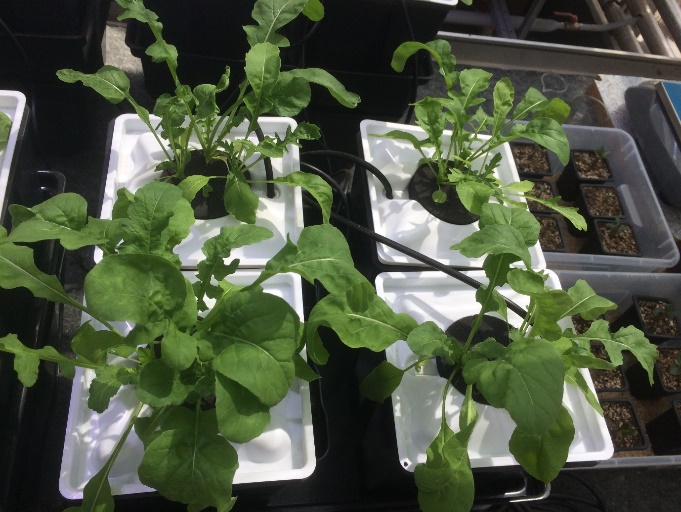


**Fig. SM2.1.** Aeroponic system AERO-STAR 40 (Platinium) with four spinach (left) and four arugula (right) plants.


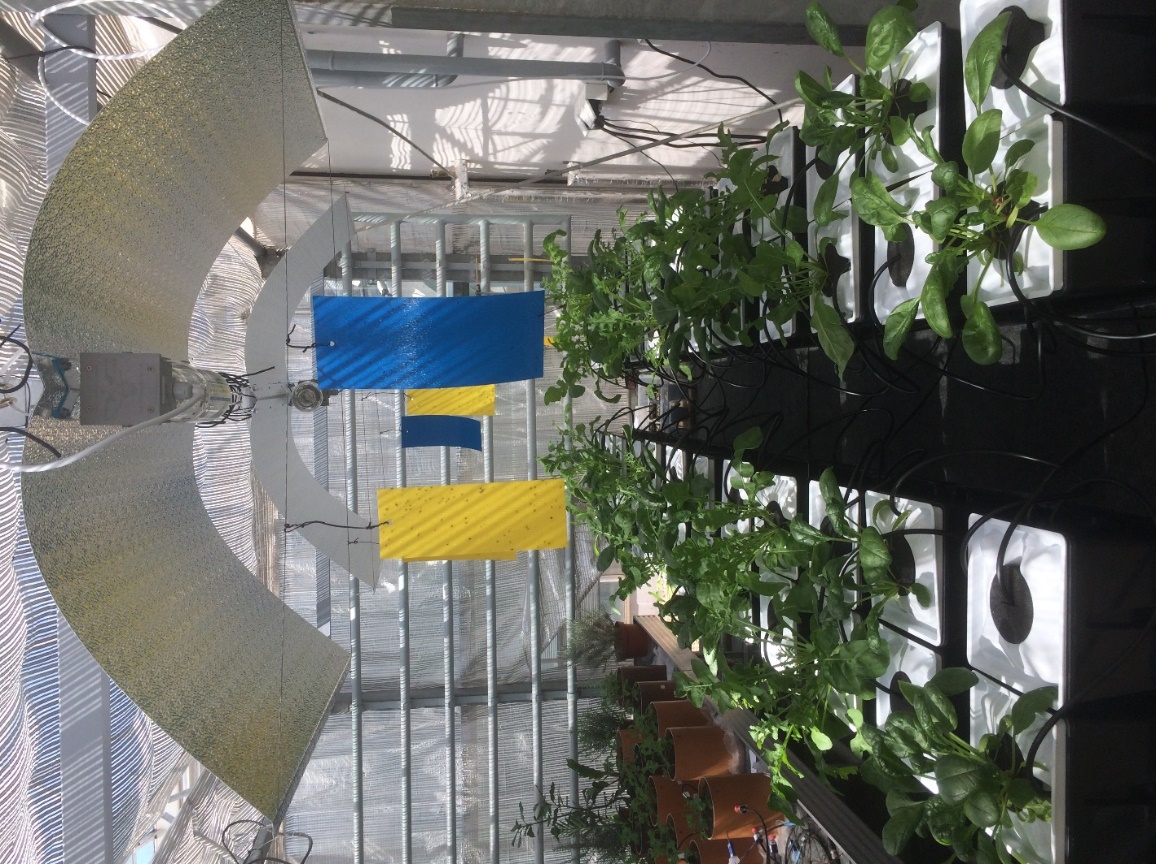


**Fig. SM2.2.** Greenhouse setup: the twelve (6 for each plant) aeroponic systems, GIB lighting Growth Spectrum Advanced lamps with a power of 600 W, and growing shades Adjust-A-Wings.


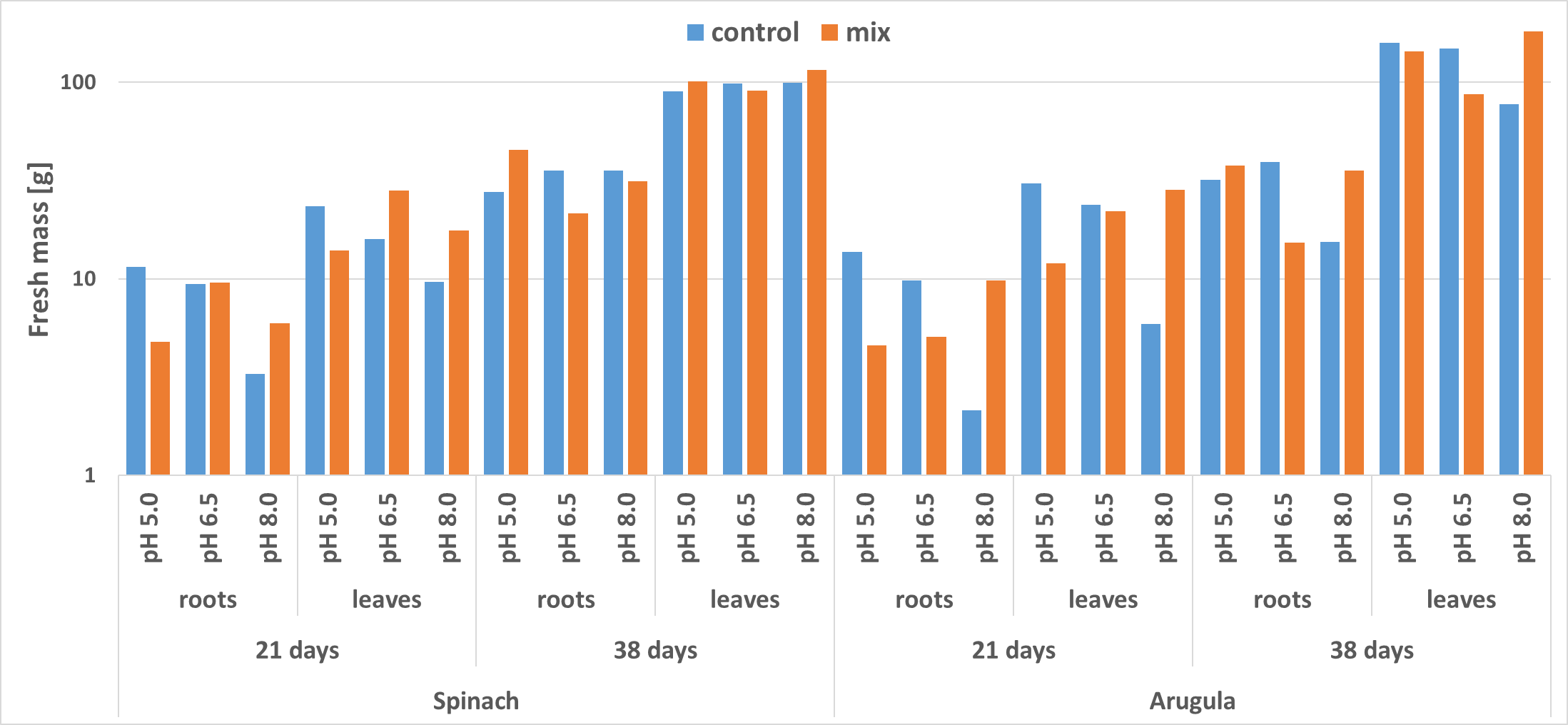


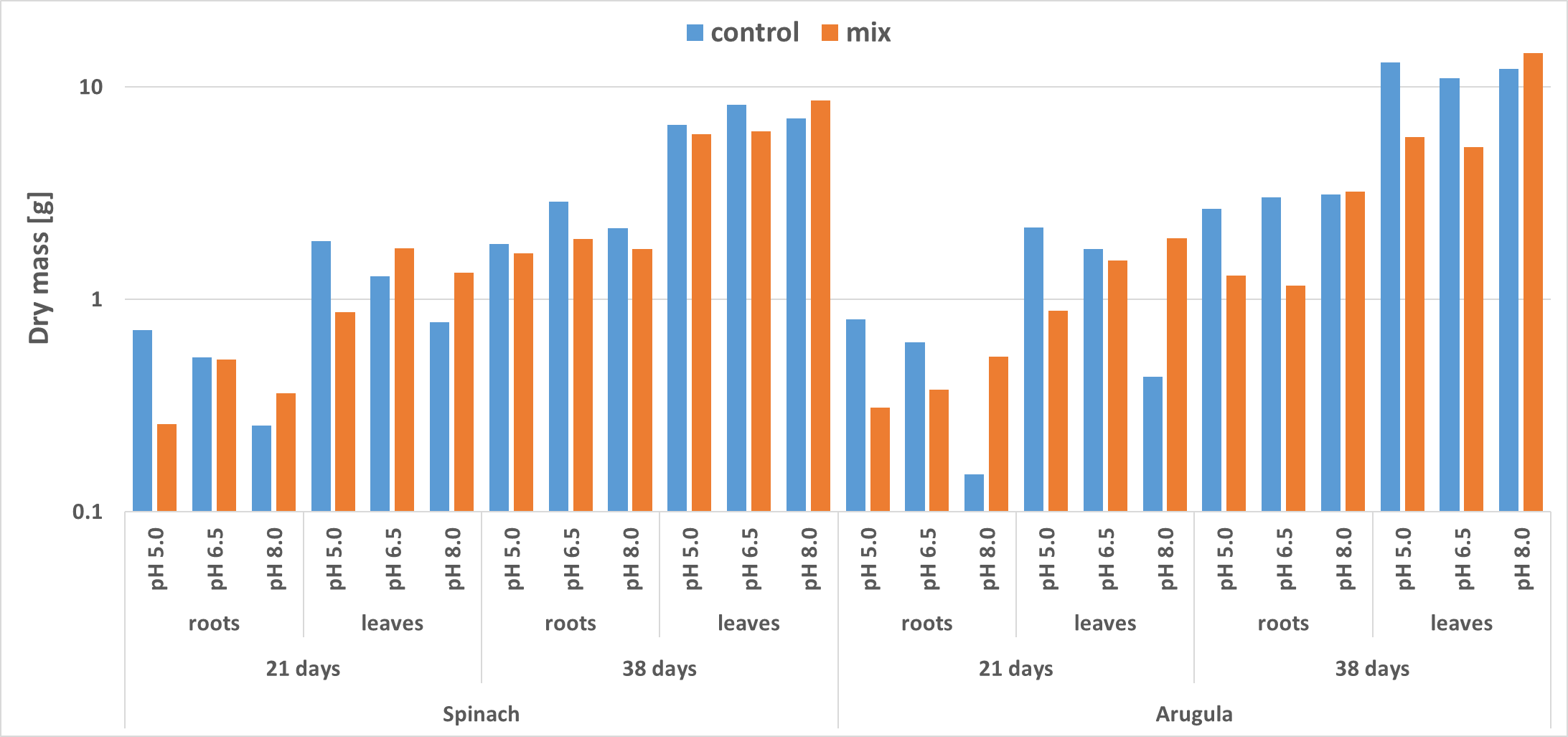


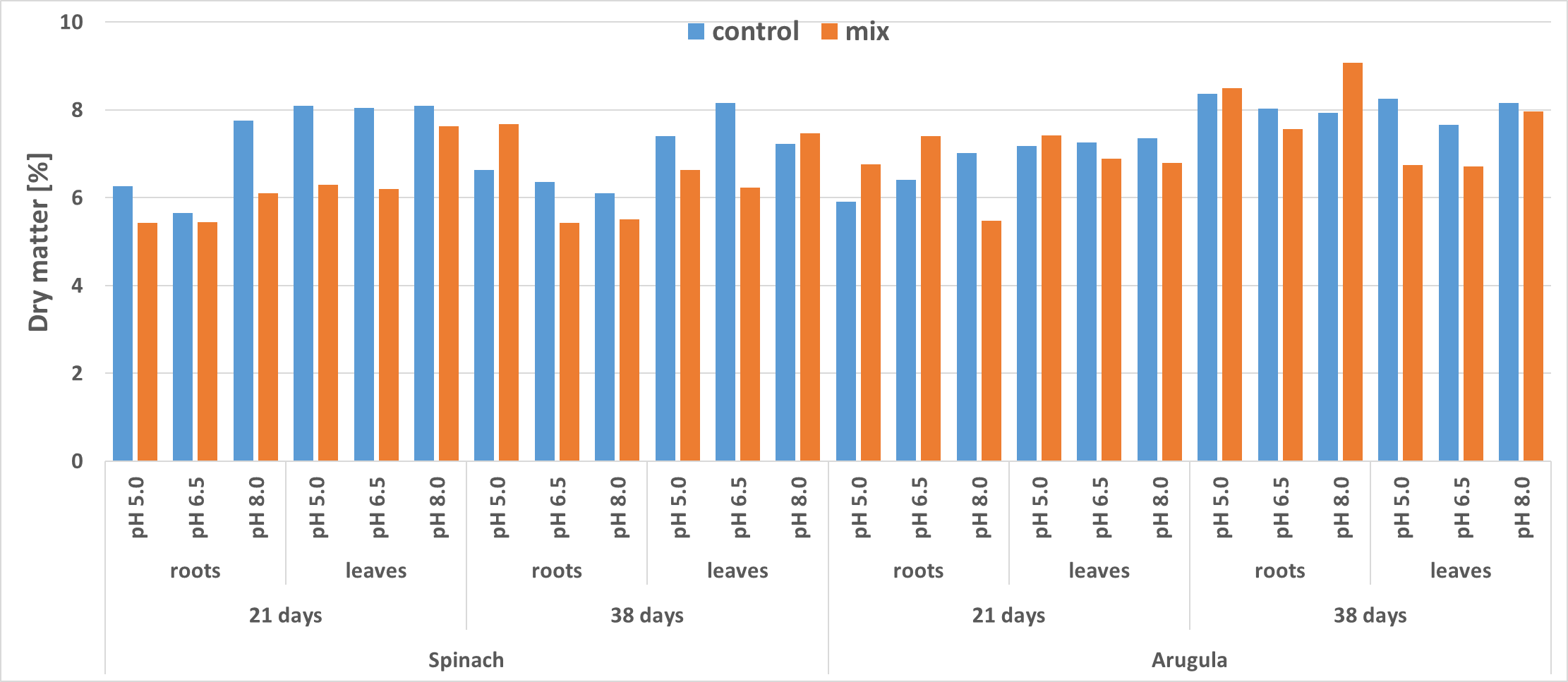
 **Fig. SM2.3.** Masses of fresh (top) and dry (middle) tissues, and percentages of tissue dry matter (bottom) of plants planted in solution without (control) and with the mixture of 3 pharmaceuticals carbamazepine, sulfamethoxazole and clindamycin (mix).


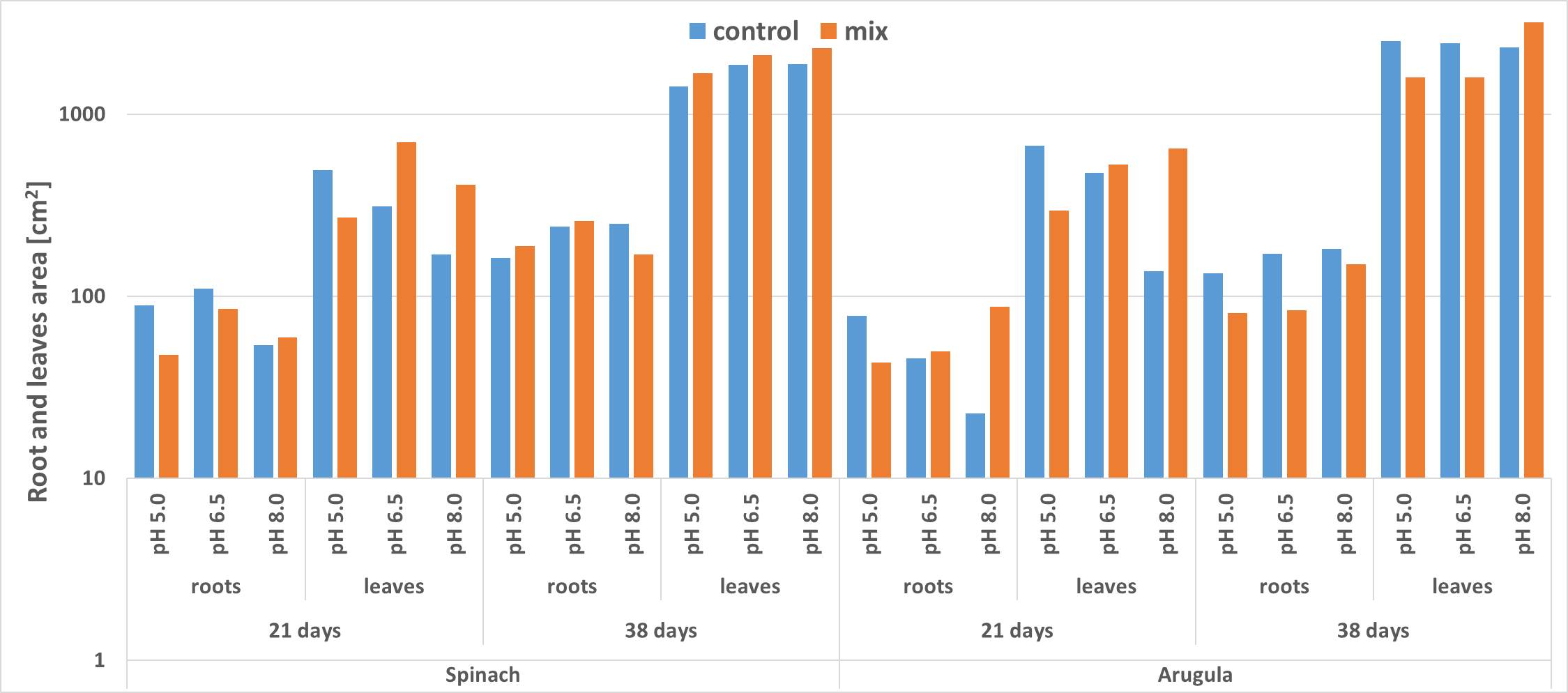


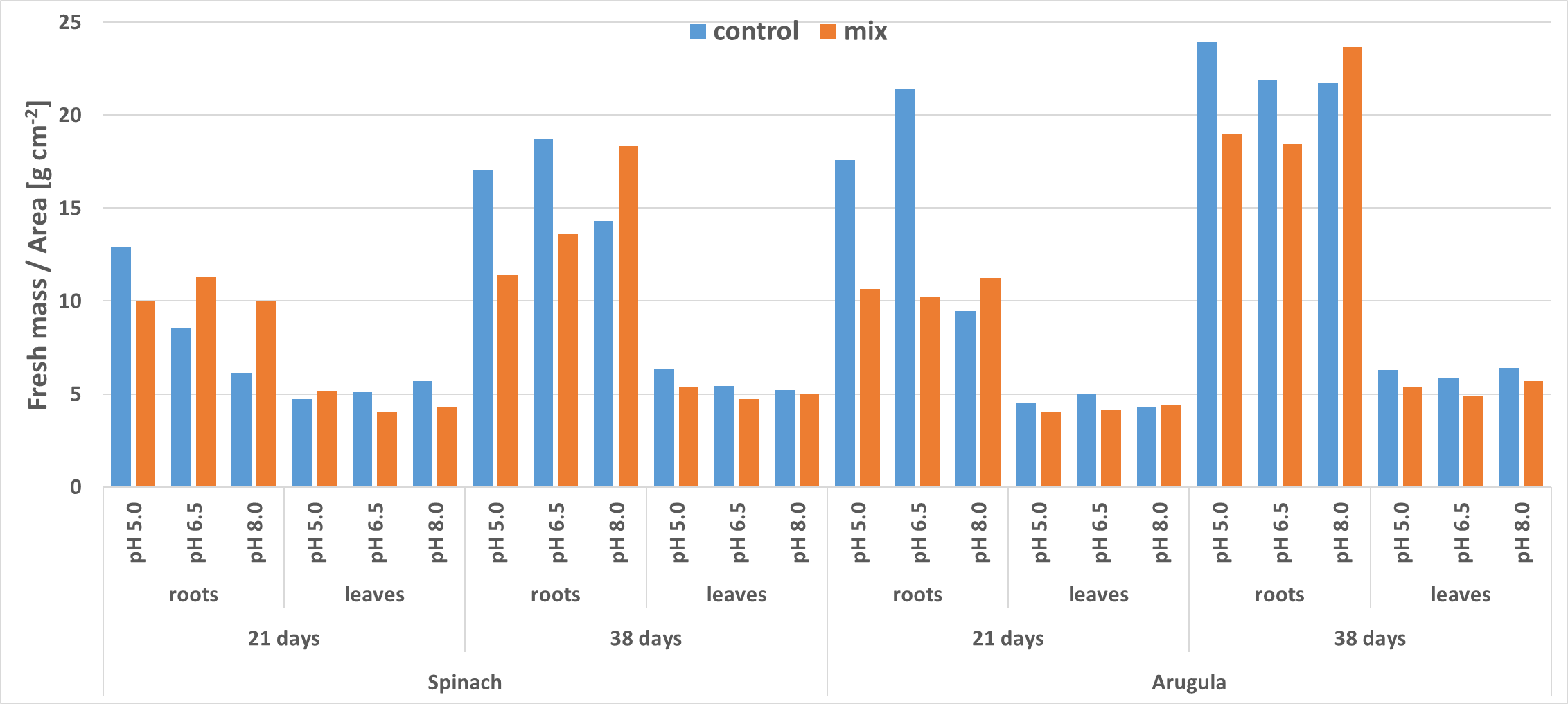


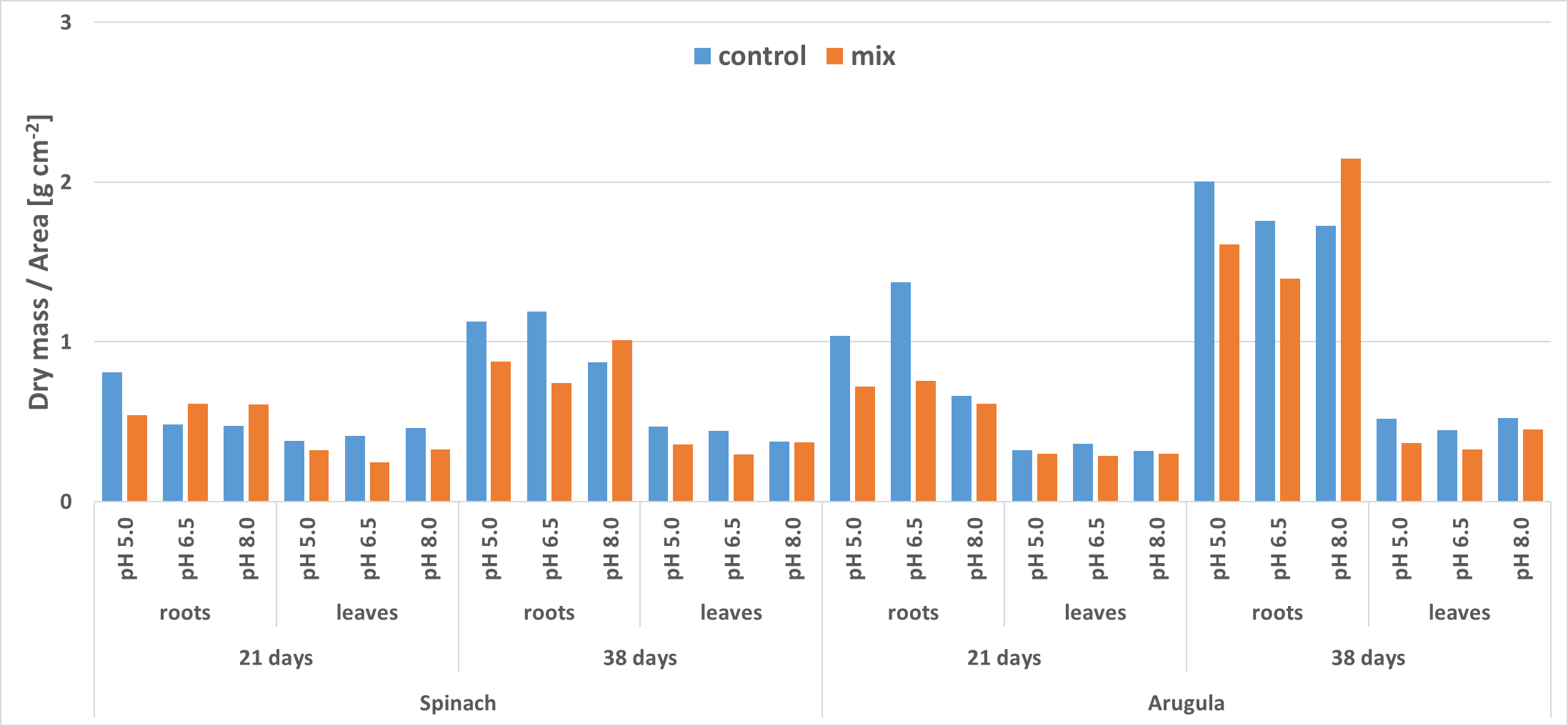


**Fig. SM2.4.** Areas of plant leaves and roots (top), masses of fresh (middle) and dry (bottom) tissues per unit area of plants planted in solution without (control) and with the mixture of 3 pharmaceuticals carbamazepine, sulfamethoxazole and clindamycin (mix).

**SM3:** LC gradient set up for separation of target analytes.

| **Time** | **Water + 0.1% FA** | **ACN + 0.1% FA** | **Flow** |
| --- | --- | --- | --- |
| **(min)** | **(%)** | **(%)** | **(μL min ^-1^)** |
| 0.00 | 100 | 0 | 350 |
| 1.00 | 100 | 0 | 350 |
| 4.00 | 75 | 25 | 350 |
| 8.00 | 40 | 60 | 450 |
| 10.00 | 0 | 100 | 450 |
| 11.50 | 0 | 100 | 450 |
| 11.55 | 100 | 0 | 350 |
| 13.00 | 100 | 0 | 350 |

FA … formic acid; ACN … acetonitrile

**HRMS conditions in positive mode:**

Capillary temperature 325°C

Spray voltage 3500 V

Sheath gas (N_2_) 40 au

Auxiliary gas (N_2_) 10 au

Isolation window (quadrupole) 1.0 m/z

Resolution (orbital trap) 15,000 FWHM

AGC target 1e6

Maximum filling time 30 ms

**SM4:** Recoveries of 18 compounds in eight different plant tissue (n=7).


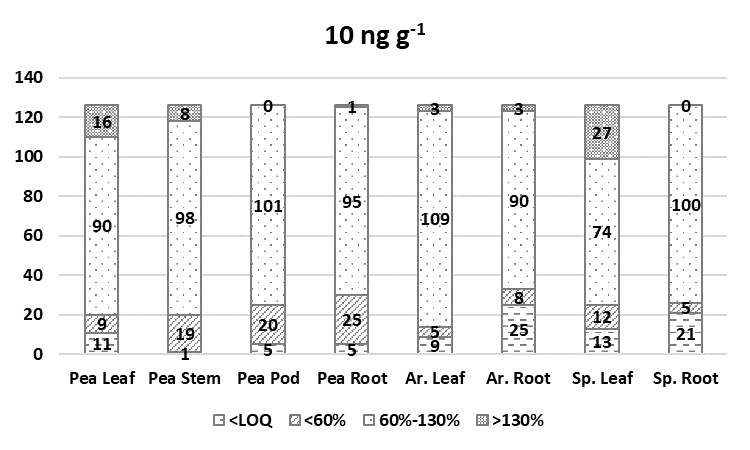


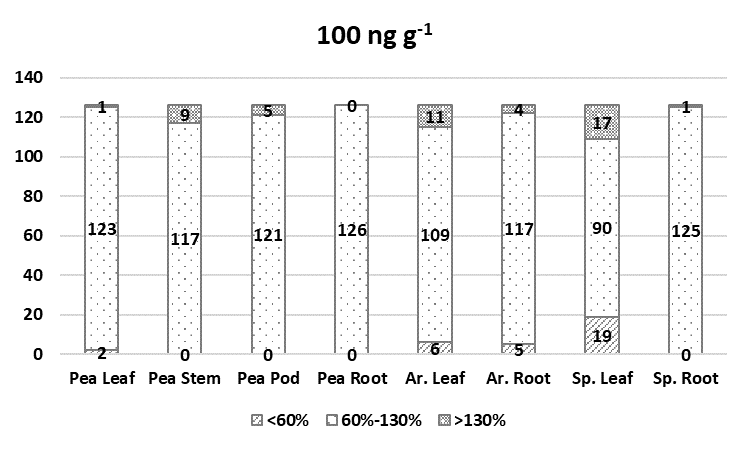

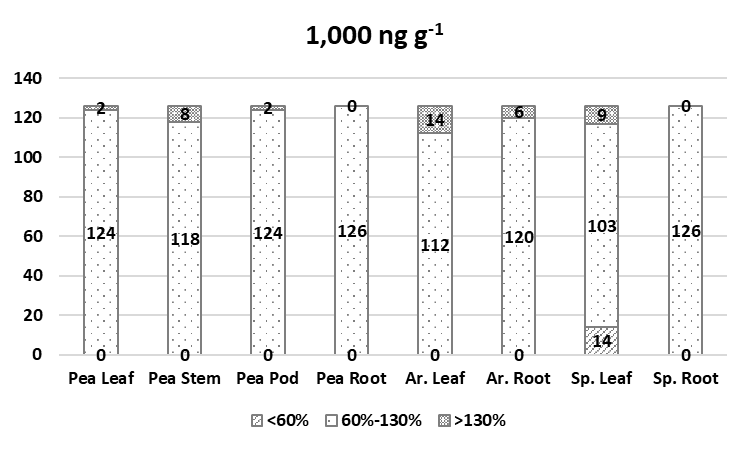


**SM5:** Matrix effects. Matrix suppression (-30%) is colored blue, and matrix enhancement (+30%) is colored in yellow.

| Compound | **Pea**  **Leaf** | **Pea**  **Stem** | **Pea**  **Pod** | **Pea**  **Root** | **Arugula**  **Leaf** | **Arugula**  **Root** | **Spinach**  **Leaf** | **Spinach**  **Root** |
| --- | --- | --- | --- | --- | --- | --- | --- | --- |
| Atenolol | 14% | 24% | 18% | 16% | 11% | 9% | 13% | 3% |
| Carbamazepine | -3% | -4% | -8% | -20% | -6% | -3% | -5% | 8% |
| CBZ 10,11 epoxide | 23% | 29% | 0% | 25% | 24% | 21% | 29% | -12% |
| CBZ dihydro | 15% | 14% | 7% | -15% | 10% | 15% | 16% | 29% |
| CBZ dihydro dihydroxy | 11% | -53% | -47% | -37% | 69% | 12% | 59% | 1% |
| Citalopram | -4% | 0% | -4% | 1% | -1% | -5% | 2% | 0% |
| Clarithromycin | -16% | -14% | -21% | -25% | -36% | -14% | 8% | -16% |
| Clindamycin | -2% | 1% | -5% | -10% | -19% | -9% | -12% | -9% |
| Clindamycin sulfoxide | 1% | -3% | 33% | 1% | -15% | -47% | -40% | 11% |
| Fexofenadine | 5% | 9% | 1% | -6% | -15% | 2% | -10% | 2% |
| Irbesartan | 4% | 3% | -2% | 2% | -4% | -6% | -1% | -2% |
| Metoprolol | 5% | 8% | 1% | 3% | -3% | -2% | 1% | -5% |
| Metoprolol acid | 38% | 38% | 50% | 34% | -50% | -15% | -8% | 13% |
| N1-Acetylsulfamethoxazole | -37% | -36% | -46% | -20% | -83% | -25% | -82% | 8% |
| N4-Acetylsulfamethoxazole | 0% | 1% | 4% | -7% | -30% | 9% | -24% | -12% |
| N-Desmethylcitalopram | 121% | 122% | 100% | 57% | -6% | -15% | -3% | -6% |
| Oxcarbazepine | 29% | 31% | 27% | 10% | -6% | 9% | 0% | -38% |
| Sulfamethoxazole | 13% | 13% | 2% | 1% | -16% | -4% | -12% | -3% |

**SM6:** Uptake of selected pharmaceuticals in spinach and arugula grown under aeroponics conditions with different pH of nutrient (5; 6.5; 8). Plants were harvested after 21 and 38 days of exposition. Concentration of pharmaceuticals are given in ng per g of dry tissue.

| Tissue | **Spinach Leaf** | | | | | | **Spinach Root** | | | | | |
| --- | --- | --- | --- | --- | --- | --- | --- | --- | --- | --- | --- | --- |
|  | 21 days |  |  | 38 days |  |  | 21 days |  |  | 38 days |  |  |
| Pharmaceutical | 5 | 6.5 | 8 | 5 | 6.5 | 8 | 5 | 6.5 | 8 | 5 | 6.5 | 8 |
| Carbamazepine | 9600 | 12000 | 11000 | 8500 | 11000 | 9400 | 2200 | 3400 | 3000 | 2000 | 3200 | 2800 |
| CBZ 10, 11 epoxide | 5200 | 7200 | 6300 | 6200 | 10000 | 8400 | 160 | 110 | 140 | 71 | 140 | 74 |
| CBZ dihydro | 14 | 6.9 | 11 | 4.5 | 7.3 | 7.9 | 1.9 | 6.5 | 1.8 | 5.5 | 3.5 | 4.2 |
| CBZ dihydro dihydroxy | <LOQ | 88 | 90 | 94 | 220 | 150 | <LOQ | <LOQ | <LOQ | <LOQ | <LOQ | <LOQ |
| Oxcarbazepine | <LOQ | <LOQ | 23 | <LOQ | 19 | 17 | <LOQ | <LOQ | <LOQ | <LOQ | <LOQ | <LOQ |
| Clindamycin | 370 | 660 | 540 | 620 | 720 | 450 | 600 | 1700 | 1500 | 800 | 3000 | 2100 |
| Clindamycin sulfoxide | 260 | 310 | 240 | 240 | 280 | 190 | 450 | 970 | 780 | 480 | 1200 | 550 |
| Sulfamethoxazole | 37 | 37 | 26 | 73 | 91 | 22 | 4100 | 3500 | 2500 | 3000 | 4000 | 2800 |
| N1-Acetyl SUL | <LOQ | <LOQ | <LOQ | <LOQ | <LOQ | <LOQ | <LOQ | <LOQ | <LOQ | <LOQ | <LOQ | <LOQ |
| N4-Acetyl SUL | 39 | 19 | 4.5 | 32 | 56 | <LOQ | 110 | 230 | 160 | 45 | 580 | 280 |
| Tissue | **Arugula Leaf** | | | | | | **Arugula Root** | | | | | |
|  | 21 days |  |  | 38 days |  |  | 21 days |  |  | 38 days |  |  |
| Pharmaceutical | 5 | 6.5 | 8 | 5 | 6.5 | 8 | 5 | 6.5 | 8 | 5 | 6.5 | 8 |
| Carbamazepine | 10000 | 14000 | 14000 | 14000 | 14000 | 14000 | 4700 | 4300 | 4700 | 4400 | 3800 | 4500 |
| CBZ 10, 11 epoxide | 730 | 1600 | 1300 | 2100 | 3000 | 3000 | 22 | 32 | 42 | 31 | 32 | 42 |
| CBZ dihydro | 16 | 25 | 40 | 23 | 28 | 38 | 5.9 | 7.6 | 10 | 2.7 | 6.8 | 5.8 |
| CBZ dihydro dihydroxy | <LOQ | <LOQ | <LOQ | <LOQ | 94 | <LOQ | <LOQ | <LOQ | <LOQ | <LOQ | 140 | <LOQ |
| Oxcarbazepine | <LOQ | <LOQ | <LOQ | <LOQ | 18 | 15 | <LOQ | <LOQ | <LOQ | <LOQ | <LOQ | <LOQ |
| Clindamycin | 810 | 1200 | 1300 | 3000 | 2500 | 4500 | 2100 | 2800 | 6800 | 7100 | 2500 | 5300 |
| Clindamycin sulfoxide | 330 | 550 | 430 | 1200 | 920 | 1400 | 690 | 1100 | 990 | 2000 | 600 | 1900 |
| Sulfamethoxazole | 81 | 71 | 54 | 100 | 42 | 110 | 4200 | 1200 | 1300 | 3200 | 1300 | 1700 |
| N1-Acetyl SUL | <LOQ | <LOQ | <LOQ | <LOQ | <LOQ | <LOQ | <LOQ | <LOQ | <LOQ | <LOQ | <LOQ | <LOQ |
| N4-Acetyl SUL | <LOQ | <LOQ | <LOQ | <LOQ | <LOQ | <LOQ | 380 | 270 | 110 | 540 | 250 | 81 |

**SM7:** Recoveries for selected pharmaceuticals in spinach and arugula from aeroponics experiment. Recoveries were estimated in triplicates at concentration level 1,000 ng g^-1^. RSD is written in brackets.

| **Pharmaceutical** | **Recovery (%)** | | | | | | | |
| --- | --- | --- | --- | --- | --- | --- | --- | --- |
|  | **Spinach Leaf** | | **Spinach Root** | | **Arugula Leaf** | | **Arugula Root** | |
| Carbamazepine | 89 | (4) | 92 | (3) | 100 | (1) | 92 | (2) |
| CBZ 10, 11 epoxide | 98 | (7) | 80 | (1) | 98 | (3) | 116 | (3) |
| CBZ dihydro | 96 | (6) | 106 | (2) | 107 | (4) | 109 | (6) |
| CBZ dihydro dihydroxy | 102 | (11) | 84 | (6) | 72 | (6) | 91 | (3) |
| Oxcarbazepine | 89 | (4) | 105 | (14) | 77 | (2) | 88 | (4) |
| Clindamycin | 106 | (2) | 104 | (1) | 101 | (2) | 101 | (3) |
| Clindamycin sulfoxide | 81 | (4) | 89 | (1) | 120 | (0) | 79 | (5) |
| Sulfamethoxazole | 123 | (4) | 119 | (3) | 123 | (4) | 109 | (2) |
| N1-Acetyl SUL | 122 | (3) | 114 | (6) | 147 | (4) | 99 | (3) |
| N4-Acetyl SUL | 103 | (4) | 100 | (5) | 113 | (4) | 111 | (7) |
|  |  |  |  |  |  |  |  |  |
